# Commentary: Effects of rhythmic auditory stimulation on motor function and balance ability in stroke: a systematic review and meta-analysis of clinical randomized controlled studies

**DOI:** 10.3389/fnins.2023.1174540

**Published:** 2023-05-05

**Authors:** Hongfei Zhao, Nanjun Ji, Wei Zhang, Jing Zhao

**Affiliations:** ^1^Department of Rehabilitation Medicine, Zibo Central Hospital, Zibo, Shandong, China; ^2^Department of Rehabilitation Medicine, Zibo Hospital of Traditional Chinese Medicine, Zibo, Shandong, China; ^3^Department of Rehabilitation Medicine, Zhangdian District People's Hospital, Zibo, Shandong, China

**Keywords:** stroke, auditory stimulation, systematic review, meta-analysis, commentary

Stroke carries a high risk of disability and is steadily increasing worldwide each year. In recent years, with the improvement of medical technology, the mortality rate of stroke patients has decreased significantly. However, most patients are left with various degrees of functional deficits, such as motor dysfunction and decreased balance (Jadavji et al., 2017). Therefore, the main goal of rehabilitation for stroke patients is to restore social activity and physical function.

Rhythmic auditory stimulation is a specific technique that promotes motor function rehabilitation by providing steady rhythmic music or monophonic beat stimulation during exercise. Rhythmic auditory stimulation promotes the rehabilitation of movements that are essentially close to biorhythmic movements (Gonzalez-Hoelling et al., 2022). The most important aspect of in these rhythmic movements is rhythmically guided gait speed and trunk control. The goal of this training is to help them adapt to their gait patterns and make a steady recovery.

We recently read an article by Wang et al. (2022) published in Frontiers in Neuroscience, which analyzed qualitatively and quantitatively the effects of rhythmic auditory stimulation on motor function and balance in stroke patients in the form of a systematic review and meta-analysis. The authors conclude that rhythmic auditory stimulation is effective in improving gait parameters, walking function and balance in stroke patients. We congratulate the authors for a very comprehensive work. However, to further improve the quality and readability of the article, we believe there are several points that could enhance the validity of these findings.

The most striking flaw of this study is the incomplete literature search. Considering that the authors did not specify too many restrictions regarding language and publication date of the literature, we searched more available databases using the given keywords. Surprisingly, two eligible studies that met the inclusion and exclusion criteria were not included (Chouhan and Kumar, 2012; Jia et al., 2017). We believe that this is due to the limitations of the databases mentioned in the text and the imperfect search strategy. Other commonly used databases such as Scopus, Google Scholar, Cochrane Library and PsycINFO should also be considered. In addition, the authors need to further optimize the search strategy by providing detailed manual search protocols in tabular form.

Another shortcoming that concerns the reader is the high heterogeneity of the results, which may cast doubt on the veracity of the findings. Possible sources of high heterogeneity include heterogeneous study quality, demographic differences in study populations, differences in interventions, inconsistent follow-up times, and diversity of control groups. In the paper, the authors used subgroup and sensitivity analyses to clarify the possible effects of the interventions. However, we suggest that the authors could have used meta-regression or subgroup analyses to discuss the other confounding factors mentioned above. To further allay the concerns from readers, we introduced an inverse variance heterogeneity (IV-het) model that could be applied to highly heterogeneous outcomes to validate the true effect size of the outcome. This model, proposed by Doi et al. (2015), can effectively mitigate the known problems of underestimation of statistical error and spurious overconfidence estimates associated with random-effects models. Therefore, we reanalyzed the three highly heterogeneous outcomes using the IV-het model. Interestingly, we found statistically significant advantages for the intervention group in terms of step length (Figure 1A), step cadence (Figure 1B) and velocity, confirming the conclusion of authors.

**Figure 1:**
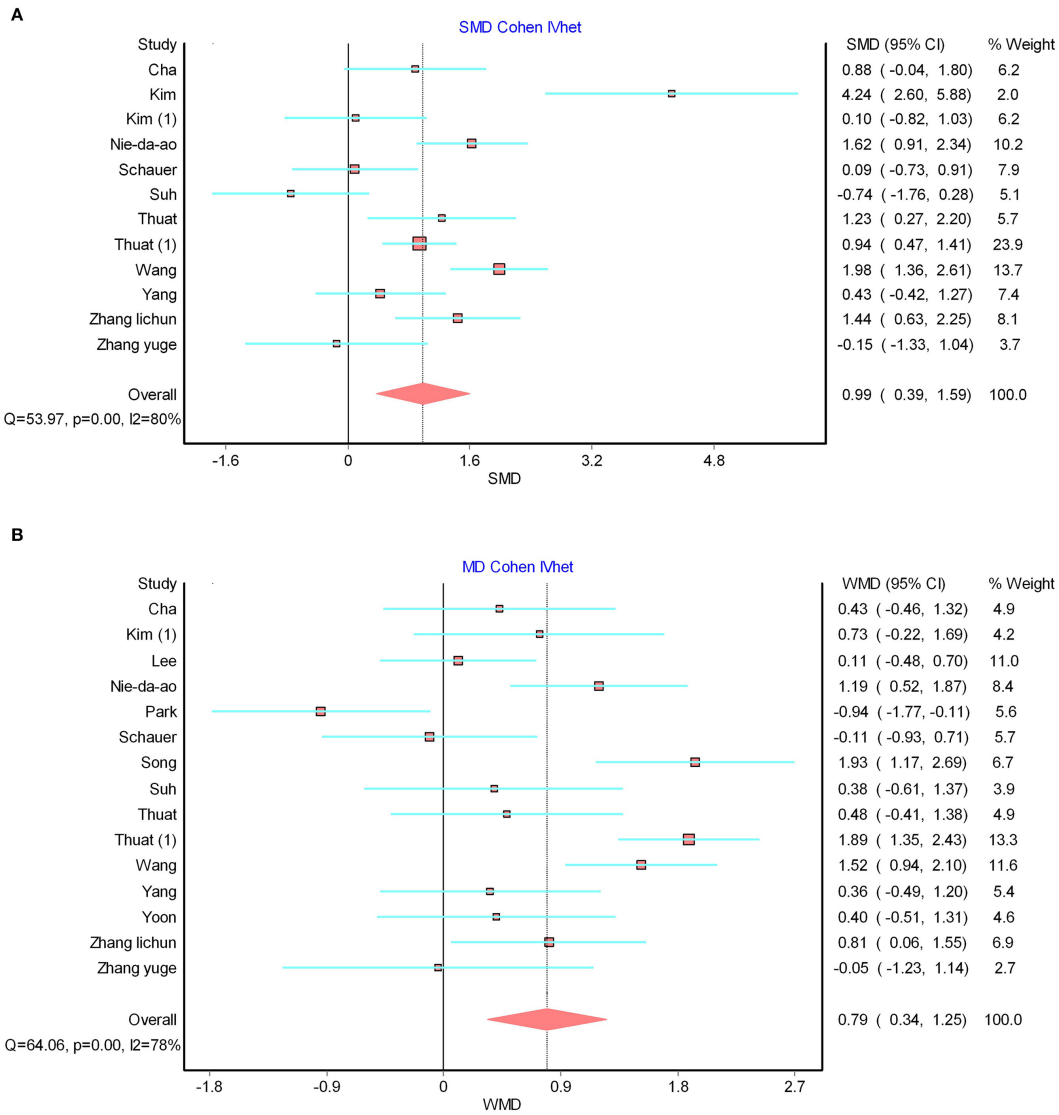
**(A)** Re-analysis of forest plots for step length based on IV-het model. **(B)** Re-analysis of forest plots for step cadence based on IV-het model.

Despite the above-mentioned shortcomings, the benefits of this intervention on motor function and balance in stroke patients still deserve recognition. The results of this meta-analysis provide solid evidence for the addition of rhythmic auditory stimulation to the rehabilitation process for a wide range of stroke patients. In future clinical practice, researchers will need to continue to monitor long-term clinical outcomes. On the other hand, the safety and efficacy of their auditory stimulation must still be ensured for an early introduction into home rehabilitation.

## Author contributions

HZ and NJ wrote the manuscript. WZ provided theoretical support. JZ reviewed the article.
